# Validation of a New Rodent Experimental System to Investigate Consequences of Long Duration Space Habitation

**DOI:** 10.1038/s41598-020-58898-4

**Published:** 2020-02-11

**Authors:** Sungshin Y. Choi, Amanda Saravia-Butler, Yasaman Shirazi-Fard, Dennis Leveson-Gower, Louis S. Stodieck, Samuel M. Cadena, Janet Beegle, Stephanie Solis, April Ronca, Ruth K. Globus

**Affiliations:** 1 KBR, NASA Ames Research Center, Moffett Field, CA USA; 2 Logyx, LLC, Mountain View, CA USA; 3 Space Biosciences Division, NASA Ames Research Center, Moffett Field, CA USA; 4 BioServe Space Technologies, Department of Aerospace Engineering Sciences, University of Colorado, Boulder, CO USA; 5 Novartis Institutes for Biomedical Research, Cambridge, MA USA; 6 LifeSource Biomedical Services LLC, Mountain View, USA; 7 Department of Obstetrics & Gynecology, Wake Forest Medical School, Winston-Salem, NC USA

**Keywords:** Microarray analysis, Ageing, Homeostasis, Translational research

## Abstract

Animal models are useful for exploring the health consequences of prolonged spaceflight. Capabilities were developed to perform experiments in low earth orbit with on-board sample recovery, thereby avoiding complications caused by return to Earth. For NASA’s Rodent Research-1 mission, female mice (ten 32 wk C57BL/6NTac; ten 16 wk C57BL/6J) were launched on an unmanned vehicle, then resided on the International Space Station for 21/22d or 37d in microgravity. Mice were euthanized on-orbit, livers and spleens dissected, and remaining tissues frozen *in situ* for later analyses. Mice appeared healthy by daily video health checks and body, adrenal, and spleen weights of 37d-flight (FLT) mice did not differ from ground controls housed in flight hardware (GC), while thymus weights were 35% greater in FLT than GC. Mice exposed to 37d of spaceflight displayed elevated liver mass (33%) and select enzyme activities compared to GC, whereas 21/22d-FLT mice did not. FLT mice appeared more physically active than respective GC while soleus muscle showed expected atrophy. RNA and enzyme activity levels in tissues recovered on-orbit were of acceptable quality. Thus, this system establishes a new capability for conducting long-duration experiments in space, enables sample recovery on-orbit, and avoids triggering standard indices of chronic stress.

## Introduction

Space travel over the past five decades reveals that multiple organ systems are influenced by novel environmental conditions above the Earth’s surface. In addition to reduced gravitational acceleration, spaceflight confers other unique environmental challenges including confinement in a closed environment, altered gas composition and exposure to space radiation, each of which has the potential to have physiological effects. Protecting humans in space requires a clear understanding of the myriad of physiologic changes that can occur in response to these exposures and their associated medical risks. As on Earth, experimentation with rodents in space enables detailed exploration of physiology and mechanisms, as well as potential mitigation strategies using experimental approaches that are not possible with humans as subjects. All but two spaceflight rodent experiments, prior to the Rodent Research (RR)-1 mission described here, have entailed sample collection only after return to Earth. In fact, some of the observed consequences of spaceflight on tissue structure^1^ and gene expression^2^ can be attributed to forces related to return and ambulation on Earth prior to sample recovery, rather than to habitation in space per se. Therefore, there is a pressing need for spaceflight hardware and operational systems to further explore the consequences of long duration space travel on rodents, while avoiding the confounding variables introduced by reentry and return of the animals to Earth.

Previous Russian Cosmos and NASA Space Shuttle missions provided multiple opportunities to perform rodent experiments up to three weeks in duration, which contributed to our current understanding of how mammals adapt to the space environment. Rodent Space Shuttle experiments conducted between 1965 and 2011 demonstrated extensive changes in the immune, musculoskeletal, and central nervous and cardiovascular systems, as well as endocrine, hematologic and metabolic changes^3^, although results between missions were sometimes inconsistent. Various independent variables such as strain, age, and duration may account for at least some of the mixed results obtained between different experiments; however, the relatively short duration of all Shuttle missions is likely to be a major factor explaining the discrepancies. Tissues adapt dynamically and at different rates to the allostatic load imposed in the course of a prolonged spaceflight experiment, which entails introduction into a novel flight hardware caging environment, launch, habitation in microgravity, then typically reentry and ambulation on Earth prior to sample recovery.

There also have been a limited number of long duration (>3 wk), rodent spaceflight experiments. A recent unmanned, Russian biosatellite experiment (Bion M1) that entailed sample recovery after return to Earth revealed that 30 days of spaceflight caused muscle, thymus and spleen atrophy (^4,5^ deficits in structure of various skeletal elements (^6,7^), altered structural morphology and changes in gene expression of several tissues^5,8^. The lengthy interval (13 hr) between capsule reentry and sample recovery for the Bion M1 mission may have affected at least some of these findings. The longest duration experiment with rodents in space completed to date (91d) in the European Space Agency’s Mouse Drawer System^9^ revealed intriguing differences in oxidative damage to red blood cells^10^ and skeletal decrements^11^, although this experiment had a limited sample size. Recently, the Japanese Space Agency performed important experiments with a newly developed flight hardware system supporting singly-housed mice, which can be centrifuged at 1 g to replace Earth’s gravity level while on the ISS^12^. Findings from their experiments reveal that 1 g artificial gravity mitigates the expected bone loss and muscle atrophy^12^ as well as retinal changes^13^ that occur after long duration microgravity. Each of the various flight hardware systems developed for long duration spaceflight has unique features, which have the potential to influence results obtained (Table 1).

**Table 1:** Rodent hardware utilized by space agencies.

Space Agency	Hardware	Maximum # of mice per chamber	Cameras	unique features	Reference
Japanese Aerospace Exploration Agency (JAXA)	MHU (Mouse Habitat Unit)	12 cages per unit; each cage accommodates 1 mouse	Yes	centrifuge compatible, smooth walls to limit ambulation, temperature sensor, crew can access animals on orbit	^12,58–62^
Italian Space Agency	MDS (Mouse Drawer System)	6 individually (max) or in groups (4 pairs)	Yes	temperature, humidity, CO_2_ and NH_3_ control system, smooth walls to limit ambulation, crew can access animals on orbit	^9,63–65^
Institute of Biomedical Problems (IBMP), Russian Academy of Sciences	Bion-M1 Block Obespecheniya Soderzhaniya (BOS)	3	Yes	temperature, humidity, CO_2_ and O_2_ controlled system, smooth walls to limit ambulation	^17,66,67^
National Aeronautics and Space Administration (NASA)	AEM (Animal Enclosure Module)	up to 10 mice or 5 rats	No	no sensors for temperature and humidity, metal grid walls, layered filter system, interior lighting, crew can access animals on orbit	^68,69^
NASA	Rodent Habitat	up to 10 mice (5 per side); 5–6 rats	Yes	metal grid walls, temperature and humidity sensors, long duration filter for improved odor containment, LED lighting (day), infrared lighting (night), crew can access animals on orbit	^14,18–24^

A brief description of rodent hardware, and its features and capabilities, utilized by the Japanese Aerospace Exploration Agency, Italian Space Agency, Institute of Biomedical Problems/Russian Academy of Sciences, and NASA.

The main goal of RR-1 was to establish new capabilities for conducting reliable and reproducible long duration experiments using rodents with on-orbit sample collection. Important objectives included monitoring animal health and welfare, and recovering samples of sufficiently high quality for global gene expression analysis using current demanding methods. The RR-1 flight experiment consisted of two separate studies with a total of 20 mice; one NASA Validation study (10 mice) and one ISS National Lab Experimental study (10 mice). The NASA Validation study was designed to evaluate the capaibilities of the rodent hardware, on-orbit operations, and sample retrieval for conducting high quality rodent expeirments consistent with current standards. The Experimental study was sponsored by the ISS National Lab on behalf of Novartis Institutes for Biomedical Research, and was designed to determine spaceflight effects on muscle atrophy in MuRF1 knockout (KO) mice and wild type (WT) mice^14–16^. Results from both studies contributed to the findings described in this paper.

The Rodent Habitat differs from other long duration spaceflight housing systems^12,17^ in that it provides for both group-housing and internal wire grating that enable grabbing and purposeful locomotion throughout the cage, much like human crew do throughout the cabin. To accommodate unique aspects of the Dragon transport vehicle and the ISS, flight hardware was modified from the Animal Enclosure Module (AEM), that was used successfully in 27 missions with rats or mice aboard the Space Shuttle^3^ followed by multiple RR missions^14,18–25^.

The RR-1 validation mission launched adult, female mice to low earth orbit in an unmanned Dragon capsule on SpaceX-4, then after 4 days the RR-1 mice were transferred to the International Space Station (ISS) where they resided for up to 33 days (total 37 days in microgravity). Female mice were selected for this mission for two reasons: first, because only female mice were flown on earlier missions that used the heritage AEM group-housed hardware system, and second, to avoid potential complications due to aggression which is more common in males than females. After this first Validation mission, pre-flight experiments were performed to optimize conditions for acclimating males in group-housed conditions^22^, and subsequently flight experiments were conducted successfully with males^19^.

Important aspects of the study design included use of adult animals, a cage system that enabled purposeful locomotion of the animals throughout the habitat, and measurements of basic indices of animal health that were compromised in past spaceflight experiments. Animal health and behavior were monitored by daily downlinked video, and samples were dissected both on-orbit by astronauts and after return to Earth from frozen carcasses. Subsequently, tissue samples were distributed to multiple scientists across the world for detailed analyses. We found mice thrived in the housing, as assessed by behavior, body weights and organ mass (adrenal, thymus, spleen). Further, samples dissected on-orbit yielded high quality RNA and protein (select liver enzyme activities). Recovery of livers dissected from frozen carcasses yielded RNA with high RIN values, although further work is needed to determine conclusively if transcriptional profiles are the same in livers freshly dissected on orbit compared to livers recovered post-flight from frozen carcasses.

## Results

### Animals

**Figure 1:**
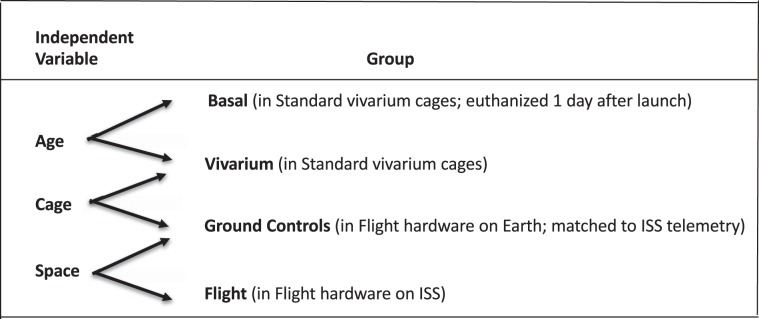
Experimental and Control Group assignments. Validation and Experimental female mice were divided into weight-matched groups as shown (n = 10/group). All groups were acclimated as described in Methods. Basal and Viv control mice were housed in standard vivarium cages, FLT and GC mice in flight hardware, and GC groups were kept in an ISS Environmental Simulator on a 4-day delay to mimic FLT conditions. A Vivarium group was not included in the Experimental study.

**Figure 2:**
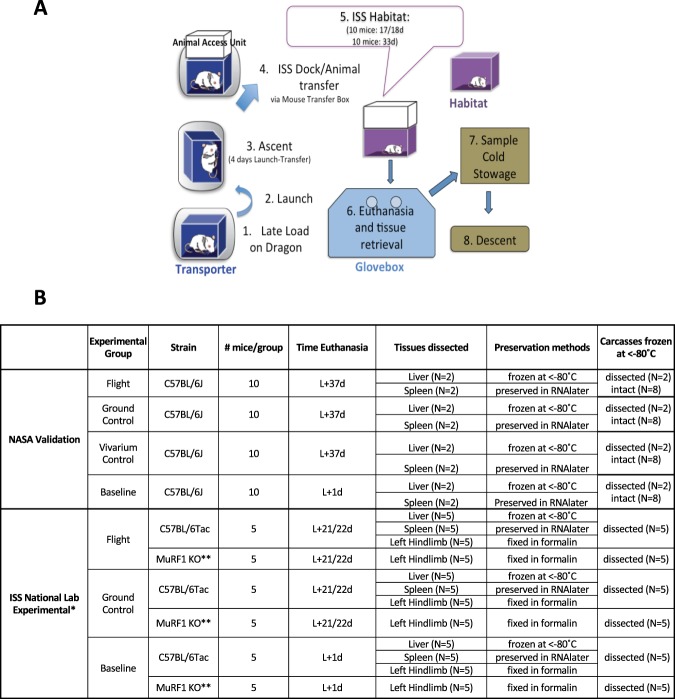
Schematic of Flight animal operations. (**A**) Flow chart of operations from launch through descent of processed tissues. Experimental (n = 10/compartment, 32 wks old at launch) and Validation (n = 10, 16 wks old at launch) mice were loaded into Transporters 2 days before launch, launched on SpaceX Dragon, then transferred into Habitats on the ISS 4 days later (5 mice/compartment). (**B**) Mice were euthanized after either 21/22 days (Experimental mice) or 37 days (Validation mice) after launch. The indicated tissues were processed for the Validation study. Spleens and livers from 5 of the 10 ISS National Lab Experimental mice were analyzed in the Validation study. *A Vivarium group was not included in the ISS National Lab Experimental study; **MuRF1 KO transgenic mice.

Mice flown on the RR-1 mission were designated as either NASA “Validation” mice (Female C57BL/6J, 16 wks) or ISS National Lab “Experimental” mice (Female C57BL/6NTac, 32 wks) to achieve the main objectives. NASA Validation mice consisted of four groups to enable assessment of the contributions of age, cage configuration, and spaceflight by comparing baseline (Basal) to Vivarium (Viv) controls, ground control (GC) to Viv, and spaceflight (FLT) to GC, respectively (Fig. 1). ISS National Lab Experimental mice included three groups: FLT, age-matched GC, and Basal groups. Animal operations and tissue processing were carried out as described in Supplementary Table S1 and depicted in Fig. 2.

C57BL/6J mice from the Jackson Laboratory were used for the Validation study since this strain has been used for previous missions on the U.S. Shuttles. For the U.S. National Lab’s Experimental study, the C57BL strain from Taconic (C57BL/6NTac) was used as the corresponding wildtype strain for the MuRF1KO mice^14^.

### Attending veterinarian observations

Health checks were performed daily through the use of downlinked videos by the Rodent Research Project Science team and the Attending Veterinarian (AV) was consulted as needed. In addition, the AV performed select audits of video to observe the animals throughout the mission. FLT and GC mice exhibited qualitatively, a typical range of behaviors, including eating, drinking, exploratory behavior, self‐and allo‐grooming, and social interactions indicative of healthy animals (also see Ronca *et al*.^18^). The extent of physical contact between FLT mice appeared less than in GC mice within a huddle (due to microgravity), although all areas of the cage were not within camera view, including space between the wall and foodbar assembly where the mice usually congregated during the light cycle, when most huddling behavior is expected to occur. FLT mice propelled themselves freely and actively throughout the Habitat using their forelimbs to push off a surface, and they quickly learned to anchor themselves using tails and/or paws as described in Ronca *et al*.^18^. The animals found spaces between the walls and the foodbar or lixits in which to rest. Spontaneous ambulatory behavior was commonly observed but did not interfere with the intake of food and water and was not deemed cause to remove any animals from the experiments.

### Spaceflight increased animal activity (validation mice)

**Figure 3:**
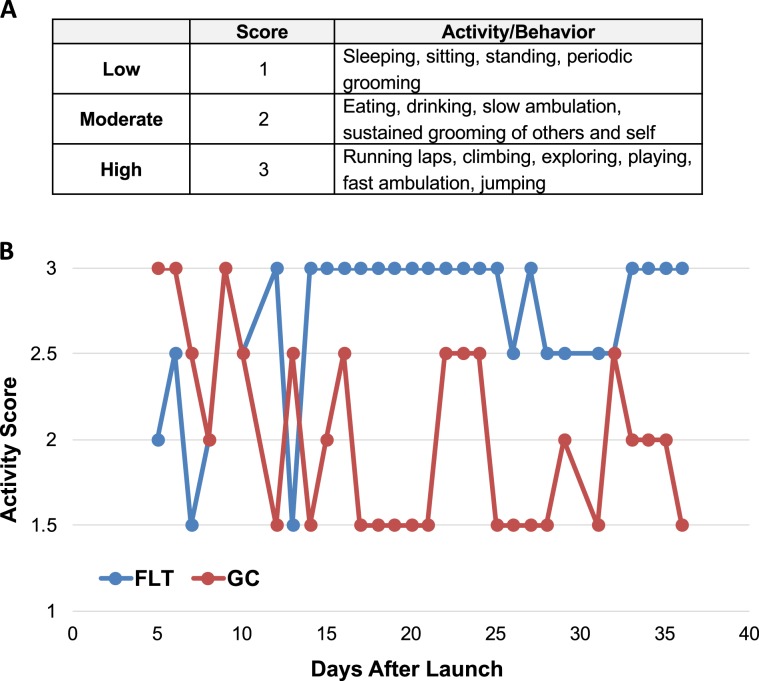
Spaceflight increased animal activity. (**A**) The scoring system used to quantify animal activity. (**B**) Relative daily activity scores for mice in each side of the Habitat for Validation Flight and Ground Control groups. N = 2 Habitat sides (containing 5 mice each) for each group of Validation Flight and Ground Control mice.

To assess animal activity, behavior on-orbit and on the ground were observed and recorded during the daily health checks. Validation FLT mice showed increased overall physical activity (eating, drinking, ambulating) in microgravity compared to Validation GC mice (Fig. 3, Supplementary Fig. S1). As expected, the animals were more active in the dark cycle than in the light cycle in all groups (Supplementary Fig. S1). The Validation FLT mice displayed a rapid and repetitive running behavior during the dark cycle that started at ~day 7–10 in microgravity, which was not observed at any time in the GC group; Experimental FLT mice also engaged in the running behavior albeit much less. No injuries of any kind were observed during the flight. Additional detailed analysis was performed to quantify and analyze on-orbit behavior and activity^18^.

### Food and water depletion

Both Validation and Experimental FLT groups showed greater food depletion compared to their respective GC groups (Supplementary Table S2). (Note that the results are described as depletion amounts rather than consumption due to the inability to discern between food and water comsuption versus wastage.) There was about twice as much water depleted in Validation FLT compared to GC (Supplementary Table S2). Similar differences between FLT and GC in food and water depletion were observed with Experimental cohorts.

### Body and tissue weights (validation mice)

**Figure 4:**
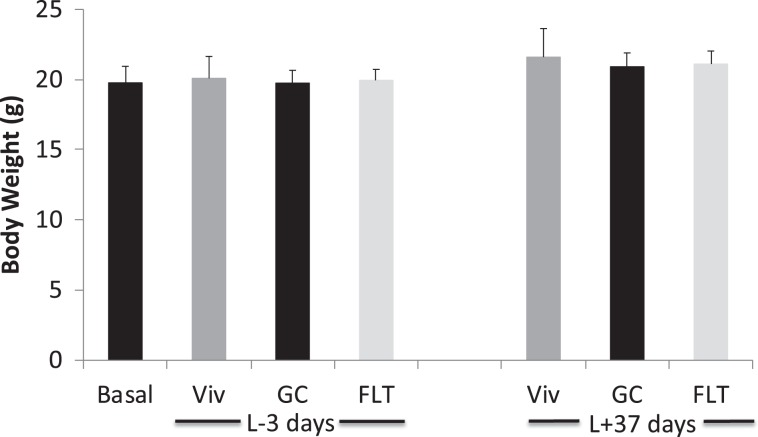
Spaceflight did not affect body weight. Mice in each group of the Validation study were weighed before launch (L-3, n = 10 for each group), and the intact carcasses that were frozen at the end of the study (L + 37 days) were thawed on Earth and weighed (n = 8 for each group). Statistical significance was determined by one-way ANOVA.

To assess general health, body weights of mice were measured twice weekly during acclimation and the final weights were measured 3 days prior to launch (L-3d) in order to select the FLT, GC, Viv, and Basal groups (as described in Materials and Methods). The body weights of Validation mice in the Basal, Viv, GC, and FLT groups did not differ 3d before launch (L-3d) (Fig. 4). There were no significant differences in body weights between any of the Validation groups at the termination of the 37d experiment (Fig. 4).

**Figure 5:**
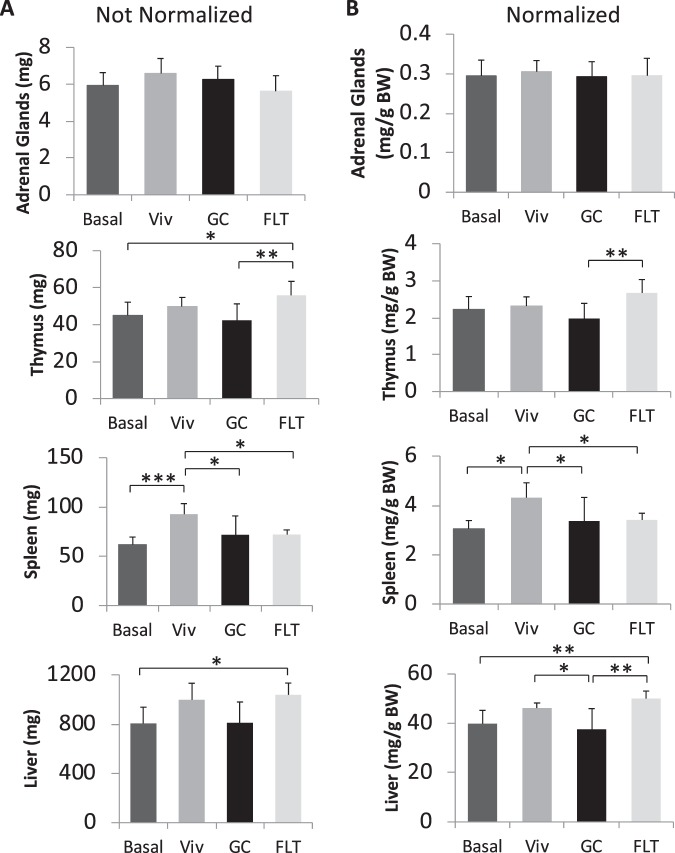
Spaceflight caused organ weight changes. After Validation FLT and control animals were euthanized, 8/10 intact carcasses per group were wrapped, rapidly frozen, and stored at −80 °C. The indicated organs were dissected from the frozen carcasses of all Validation groups, thawed and weighed. Raw weights of each organ (**A**), and organ weights after normalization to body weight (**B**) were quantified. N = 8 per group (FLT, GC, Viv, and Basal) for adrenal and thymus weights; n = 8 per group for spleen weights (except for the FLT group, n = 7); n = 7 per GC group and n = 6 per FLT and Viv groups for liver weights. *p < 0.05; **p < 0.005; ***p < 0.001. Statistical significance was determined by Levene’s test for uniformity of variance and Shapiro-Wilk test for normality goodness of fit followed by one-way ANOVA. *Indicates significant differences by Tukey-Kramer HSD post hoc test.

In addition to total body weight, the following muscles and organs were dissected from thawed Validation carcasses and weighed: gastrocnemius, soleus, tibialis anterior (TA), extensor digitorum longus (EDL), quadriceps, liver, spleen, thymus, and adrenal glands. The raw weights of thymi were significantly different between FLT and GC animals; FLT thymus glands were ~32% higher than GC. (Fig. 5A).

The thymus glands normalized to body mass weighed more in FLT mice than GC (by 35%), as did the livers (by 33%)(Fig. 5B). The normalized weights of the adrenal glands between the two groups did not significantly differ (p = 0.09) (Fig. 5B).

**Figure 6:**
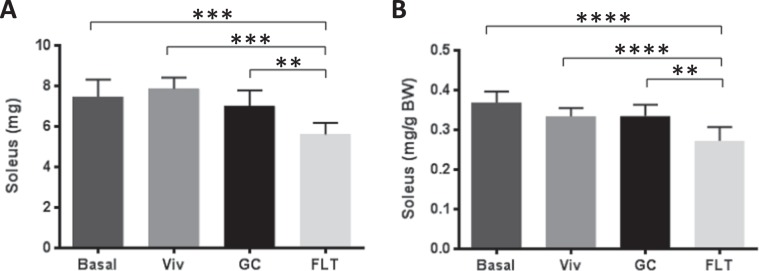
Spaceflight reduced soleus mass. The soleus was dissected from the frozen carcasses of all Validation groups, thawed and weighed according to methods. Raw soleus weights (**A**), and soleus weights after normalization to body weight (**B**) were quantified. Data are shown as mass per both hindlimbs (average). N = 8 for each group. *p < 0.05; **p < 0.01; ***p < 0.001. Statistical significance was determined by Levene’s test for uniformity of variance and Shapiro-Wilk test for normality goodness of fit followed by one-way ANOVA. *Indicates significant differences by Tukey-Kramer HSD post hoc test.

The soleus muscle weight was reduced by ~19% in Validation FLT versus GC animals (Fig. 6). Of all the muscle tissues analyzed (gastrocnemius, soleus, tibialis anterior, EDL, and quadriceps), the soleus was the only one that showed significant differences in FLT compared to GC (Supplementary Fig. S2).

Differences between age-matched control groups indicating cage effects also were observed. The spleens and livers from GC animals weighed less than Viv controls by 22% and 19% respectively (Fig. 5B).

### Quality of sample return from spaceflight animals

To test preservation quality, three approaches were used following on-orbit dissection. (1) freezing (liver) (2) preservation in RNAlater (spleen), or (3) freezing carcasses on-orbit followed by dissecting tissues and organs from frozen carcasses after return to Earth. To determine if these methods were sufficient to achieve high quality tissue samples, liver samples were analyzed for select enzyme activities and total RNA was extracted from liver and spleen samples (as described in Materials and Methods) and RIN value obtained as an indicator of RNA quality.

**Figure 7:**
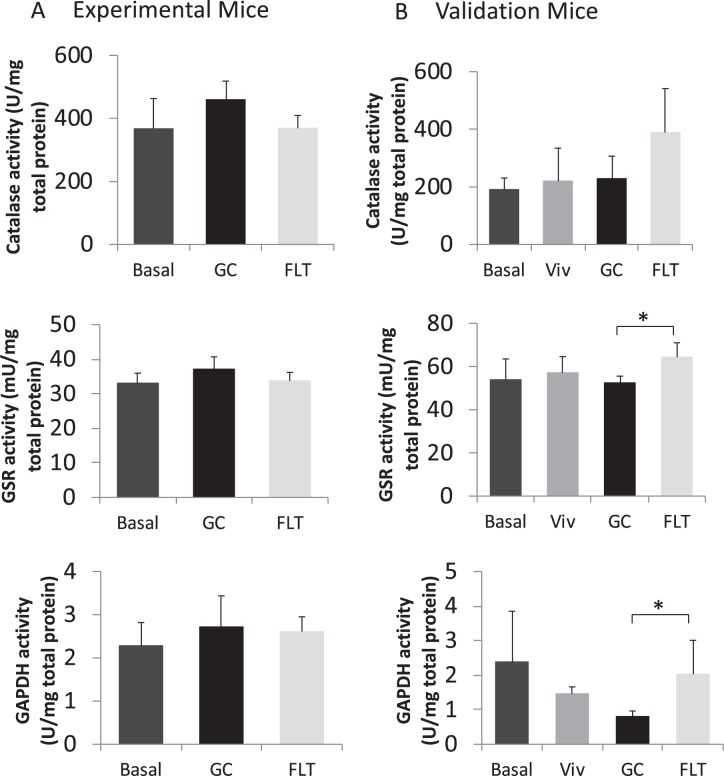
Spaceflight increased liver GSR and GAPDH activity. Livers were dissected after 21/22 days in microgravity and frozen at −80 °C until recovery (Experimental mice, **A**), or were recovered post-flight, after 37d, from frozen carcasses (Validation mice, **B**). Tissue lysates were analyzed for liver enzyme activities. N = 4–5 for each group. Significant main effect by comparing age-matched groups for GSR at P < 0.0256 and for GAPDH at P < 0.0147. *Indicates significant difference between FLT and GC. Statistics were performed without the inclusion of Basal values. For catalase activity, statistical significance was determined by the non-parametric Kruskal-Wallis test. GSR and GAPDH activities were analyzed by one-way ANOVA followed by Tukey-Kramer HSD post hoc test.

Activity levels of the enzymes Catalase, Glutathione reductase (GSR), and Glyceraldehyde 3-phosphate dehydrogenase (GAPDH) in all groups of the Validation and Experimental mice were measured (Fig. 7). No differences between groups were observed in the Experimental mice. There were no significant differences in GSR levels when all four groups of the Validation mice were compared. GAPDH activity levels also were not significantly different between GC and FLT groups in Validation mice. However, comparing the age-matched groups (i.e. Viv, GC and FLT), GSR and GAPDH activity levels were elevated significantly in FLT mice compared to GC mice (23% and 74% respectively), but not compared to Viv controls (Fig. 7). Catalase activity in Validation FLT mice appeared elevated compared to GC and Viv controls, but showed only a trend for a main effect difference (P = 0.0846, Kruskal-Wallis test). Similarly, catalase protein content appeared elevated in FLT compared to GC, but comparison of the age-matched groups showed only a trend for a main effect difference (P < 0.0621, ANOVA, data not shown). Catalase specific activity (I.U./μg catalase protein) was not different between the groups (data not shown). In addition, activity levels of these enzymes were comparable to values obtained for samples collected as positive control samples (PC), which had been dissected immediately after performing euthanasia on the mice and then rapidly frozen in liquid nitrogen (data not shown). Thus, both on-orbit recovery and frozen carcass recovery did not markedly deplete activity levels.

**Figure 8:**
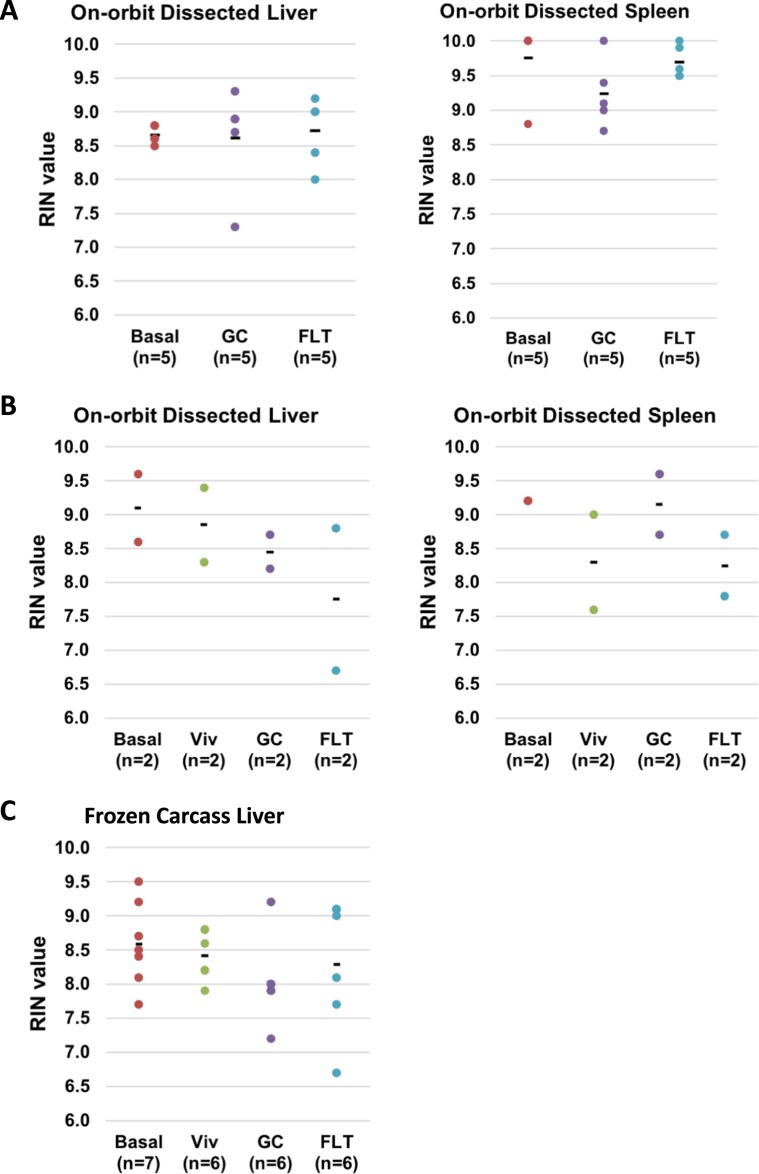
RNA quality from from livers and spleens dissected on-orbit and/or from frozen carcasses. RNA Integrity Numbers (RIN) were determined to assess RNA quality as described in methods. RNA was extracted from livers and spleens of (**A**) Experimental mice and (**B**) Validation mice that were dissected and preserved on-orbit (or on the ground) as described in Methods. (**C**) RNA was extracted from livers that were dissected from Validation carcasses that had been frozen intact on-orbit (or on the ground) and stored at −80C until the time of dissection. N values are indicated for each group.

The RNA extracted from spleens of Experimental FLT mice which had been dissected on-orbit then preserved in RNAlater, and from livers dissected on-orbit then immediately frozen, had RIN values > 8.0 (Fig. 8A), which is generally considered sufficient for demanding molecular analyses such as RNA sequencing. The RNA extracted from the Validation FLT animal livers (that were dissected on-orbit then immediately frozen) and spleens (dissected on-orbit and preserved in RNAlater) had RIN values, >6.5 and >7.6, respectively (Fig. 8B). RNA extracted from liver tissues dissected from the frozen carcasses of Validation FLT mice had RIN values > 6.5 (Fig. 8C).

### Liver gene expression analysis (validation mice)

To begin to assess gene expression in samples recovered from frozen carcasses (FC, n = 5/group) and on-orbit dissected tissue (OoD, n = 2/group), total RNA was analyzed by RTqPCR for expression of 12 liver-associated genes: *Hmgcr, Cat, Gsr, Serpina1e, Cdkn1a, Chka, Pnpla3, Tef, Snd1, Nr4a1, Nox4*, and *Taf*. These specific genes were selected for analysis on the basis of prior spaceflight findings^26,27^ and preliminary bioinformatics of RNAsequencing results (Homer Fogle and Jonathan Galazka, personal communication). In frozen carcasses, expression of *Gsr*, *Cdkn1a*, and *Chka* were elevated in FLT mice compared with GC mice, and *Tef* expression was reduced as a result of spaceflight (Supplementary Fig. S3). Patterns of relative gene expression in samples recovered from frozen carcasses of FLT vs GC mice from the Validation study did not appear to be the same in samples recovered from on orbit dissected tissues. Although the small sample size from on orbit dissected tissues precludes drawing definitive conclusions, these prelimary results indicate that the method of sample recovery may have affected RTqPCR results obtained.

## Discussion

The main objective of this work was to develop new capability for conducting long duration, rodent experiments in space which both minimize the complications of non-specific, chronic stress responses and allow for sample recovery on-orbit, thereby avoiding the confounding variables associated with returning to Earth. All animals were healthy throughout the duration of the mission as evidenced by daily video health checks, maintenance of body weights compared with ground controls, and an absence of immune organ atrophy (thymus, spleen) or adrenal hypertrophy relative to ground controls. As expected, the mass of the soleus, consisting predominantly of type I muscle fibers which typically atrophy with disuse, was significantly lower in Validation FLT mice than in GC mice housed in the flight hardware (Fig. 6), whereas other muscles did not. Further, we found that all of the forms of tissue collection/preservation tested (on-orbit dissection followed by freezing, preservation in RNAlater, or freezing carcasses on-orbit followed by dissecting tissues and organs from frozen carcasses after they were returned to Earth) provided samples with high enough quality for molecular analyses, although the tissue collection/preservation methods may have affected results obtained. Taken together, these results demonstrate a new capability for long duration experimentation with on-orbit sample recovery.

Body weight is considered a sensitive indicator for monitoring animal health. We found Validation FLT mice had similar body mass as a consequence of spaceflight when compared to GC and Viv mice (Fig. 4). This finding is in contrast to previous reports from numerous rodent spaceflight experiments^28^. Some previous spaceflight experiments have on occasion shown that spaceflight can lead to reduced body weights compared to baseline values and ground controls^29,30^, complicating interpretation of the results obtained. If the body weight loss is due to persistent stress response entailing activation of the HPA (hypothalamic, pituitary and adrenal) axis, then it can be difficult to distinguish between effects of reduced gravity-dependent loading in space vs. endocrine stress. These findings, together with similar findings from tissue weights of adrenal gland, thymus and spleen and behavioral observations, are consistent with the interpretation that habitation on-orbit did not provoke a chronic stress response.

Both the Validation and Experimental FLT mice engaged in more active behaviors than their respective GC mice (Fig. 3, Supplementary Fig. S1). This finding was surprising but consistent with a recent report that simulated weightlessness (14d) also leads to increased locomotion relative to ambulatory controls after a transient initial period of reduced motor activity^31^. Furthermore, the Validation FLT mice exhibited a rapid, repetitive running behavior, which was not observed in the Experimental mice (Fig. 3 and Supplementary Fig. S1). This difference in behavior between the cohorts was likely due to their age differences (the Validation mice were 16-weeks old at launch, whereas the Experimental mice were 32-weeks at launch). A detailed behavioral analysis from this mission can be found in Ronca, *et al*.^18^.

Atrophy of the slow-twitch soleus muscle, which provides postural support during mechanical loading, occurs as a consequence of spaceflight in rodents^12,32^. Consistent with previous studies, we observed a ~19% decrement in soleus weight in Validation FLT mice compared to GC (Fig. 6), but no differences in the EDL. Despite previously reported atrophy of the gastrocnemius muscle due to microgravity^12,29,30,33^, no difference in gastrocnemius mass was observed in Validation FLT versus GC mice (Supplementary Fig. S2). The lack of atrophy in the gastrocnemius was especially surprising since C57BL/6 female mice (9 weeks old at launch) utilized in previous space shuttle experiments^29,30^ housed in very similar hardware to that used in this study, showed significant atrophy of gastrocnemious, soleus and tibialis anterior. Interestingly, these mice were shown to lose body mass with spaceflight compared with basal controls despite their relatively young age and expected growth pattern. A possible explanation for the differences in gastrocnemius findings between these previous space shuttle investigations^29,30^ and the current study is that young, growing mice at time of launch may be more sensitive to spaceflight effects than older (Validation) mice. Alternatively, perhaps mice are less active during initial exposure to spaceflight, leading to muscle atrophy, and then become more active over time, which in fact was found to be the case by Ronca *et al*.^18^. In a longer duration experiment such as Validation RR-1, indices of atrophy may reverse. In any case, the finding that there was reduced soleus muscle mass in the Validation FLT mice, as well as the Experimental FLT mice^14^ was consistent with expected findings of slow-twitch muscle atrophy due to long duration spaceflight.

With respect to organ weights, thymus glands and livers were significantly heavier in FLT mice compared with GC mice (Fig. 5) whereas spleen weights did not differ. Typically, shorter duration experiments than the Validation study show that spaceflight causes atrophy of the immune organs, which is often attributed to an endocrine stress response, although direct effects of microgravity on immune cells *in vitro* also occur^34^. The greater thymus weights observed in FLT animals relative to GC contrasts to observations from previous, short and long term missions^27,35–37^, but is consistent with findings from other spaceflight experiments. Unlike previous missions, tissues from mice analyzed in the present study were preserved on-orbit, and therefore the animals did not endure the added challenges of a return flight to Earth and ambulation, which can potentially affect gene expression as well as soft tissue weights^38^.

Livers from Validation FLT mice had a 33% greater mass than GC controls (when normalized to body weights), a finding consistent with only some prior spaceflight studies^27^. Jonscher *et al*.^39^ reported increased accumulation of hepatic lipid droplets and increased total triglycerides in spaceflight mice livers compared with livers from GC mice and Blaber *et al*. describe impaired oxidative defence pathways due to spaceflight^26^. Early, short duration rat spaceflight experiments showed that spaceflight increased hepatic lipid peroxidation, altered enzyme protein levels^40,41^, and together with later studies, led to the proposal that spaceflight leads to pathological changes consistent with the eventual development of non-alcoholic fatty liver disease with long duration spaceflight^39^. The relevance of spaceflight-induced liver toxicity in rodents to human astronauts remains uncertain. Unlike musculoskeletal and immune effects of spaceflight which are observed in both humans and rodents, to our knowledge there have been no reports showing direct evidence that liver function is altered in astronauts during missions lasting up to one year.

In Validation mice, spaceflight increased activity levels of GSR and GAPDH, relative to cage-matched GC. GSR plays a critical role in resisting oxidative stress and spaceflight can induce ROS-related activity^27,42^. Our findings that GSR activity levels were elevated in FLT mice relative to GC contrasts to those of Hollander *et al*.^43^, who shows reduced hepatic GSR activity in rats after an 8d flight along with a decline in other antioxidant enzymes and increased malondialdehyde, consistent with a state of oxidative stress. The differences in GSR activity between Validation (33d on-orbit) versus Experimental (17–18d on-orbit) mice, as well as rats (8d)^43^, may be due in part to the length of spaceflight exposure although other explanations certainly are possible (e.g., age). GAPDH plays a principal role in glycolysis and gluconeogenesis. Although GAPDH is commonly considered a housekeeping gene and applied as a control for gene or protein expression levels, it is in fact a redox-sensitive protein regulated at the transcriptional level by insulin and hypoxia^44^ and also in the liver during periods of nutritional insufficiency^45^. Further, short duration shuttle missions lead to reduced rather than elevated expression of glycolysis-related genes in the liver^46^. Together, these findings suggest time-dependent regulation of oxidative defense and glycolytic pathways during spaceflight, which will require further study in future time-course experiments.

Enzymatic activity can be a good indicator of the protein quality following collection and preservation, and therefore, we measured activity levels of select enzymes from livers dissected and frozen on-orbit by astronauts and those dissected from frozen carcasses after return to Earth in comparison to control samples collected on Earth (positive controls). GC sample collection and storage was conducted to duplicate the on-orbit procedures (including dissection timing, preservation and storage) with the highest fidelity possible. Enzyme activities from livers of Experimental FLT mice that were dissected and preserved on-orbit and from livers of GC mice, were similar to those of samples prepared under optimal laboratory conditions, as well as protocols for GC sample collection and storage that were specifically planned to duplicate on-orbit procedures, including dissection timing and preservation.

The RNA extracted from livers that were dissected then immediately frozen on-orbit were compared with those dissected from the frozen carcasses. RNA from livers, either freshly dissected or from carcasses, exhibited RIN values that exceeded 6.5 (Fig. 8). RIN values for spleens that were dissected then preserved in RNAlater (Fig. 8C) exceeded >7.5. RNA recovered from frozen spleens in preflight experiments consistently yielded RNA with very low RIN values, possibly due to higher RNase expression in this organ (results not shown); therefore this approach for sample recovery was not used on the flight samples. Taken together, findings from the liver tissue indicate that both methods of spaceflight tissue preservation were sufficient to yield RNA with RIN values generally considered adequate for most molecular analyses although possible differences in patterns of gene expression assessed by RTqPCR require further evaluation to determine if recovery from frozen carcasses yields unreliable results, perhaps due to warm ischemia^47^ in liver samples recovered from the carcasses (Supplementary Fig. 3). Studies are in progress to further assess this possibility.

Conducting spaceflight experiments presents unique challenges to making rapid progress in understanding the biological responses of rodents to microgravity. Not the least of these challenges is ensuring that control animals on Earth are provided a physical environment that replicates the environment on the spacecraft to the greatest extent possible. One limit of this study, as opposed to some prior spaceflight missions^48–50^ is that ground controls were not subjected to the vibration, acceleration and noise stimuli which is unavoidable during launch of rodents into space. This was due to logistical and cost limitations imposed on this system. We reasoned, though, that because the system was intended to support long duration experiments, the effects of transient stressors associated with launch would diminish by the time of sample recovery. Further study is needed to justify this expectation, especially for those tissue responses which are most likely to be sensitive to the stress of launch. Further, unique habitats and environmental control systems must be devised to adequately support both FLT and GC animals. The hardware developed to transport and house the mice did in fact display differences; we found GC values for several parameters were different than Viv, as others also have found using the heritage habitat, AEM, on Shuttle missions (e.g.^43,51^). While comparison of FLT mice to the cage-matched GC group is clearly of greatest relevance for determining the influence of spaceflight, the Viv control group is also useful for comparing results obtained in a given spaceflight experiment to standard housing conditions in investigators’ home institutions.

In conclusion, on the basis of our findings and those of others, we propose that physiological

accommodation to the spaceflight environment entails at least a two-phase process. An initial acute (<2–3 wk) phase yields rapid and pronounced differences in tissue biology, which are likely to be dynamic, transient, and sensitive to the acute psychological and physical stress of launch, and adaptation to the novel microgravity environment. Evidence for an early, transient phase of adaptation is based on findings from previous spaceflight experiments that often show spaceflight causes decrements in body mass, immune organ atrophy, and indices of HPA axis activation after missions lasting less than 3 wk^12,28–30,52,53^. We suggest a second, long duration (>2–3 wk) phase follows when the animals have acclimated to reduced loading microgravity; our findings that body weights, immune organ mass and adrenal mass were not lower in Validation spaceflight mice compared to ground controls or basal groups support this proposal. Return to earth would be expected to introduce a new phase when the effects of gravity-dependent loading exceeds the new steady-state achieved during residence in microgravity. Since the first mission described here, the hardware system and operations for this project were developed further to support return to Earth of living animals, enabling study of the entire cycle of long duration space travel. Although there are individual variabilities, similar phases of adaptation to spaceflight are seen in crew members.

## Methods

### Hardware description and performance

The Rodent Research Transporter and Habitats performed as expected for the duration of the flight. There were no interruptions in power, lighting, airflow, or the delivery of food and water throughout the duration of the mission. Lights were programmed on a 12:12 hr light/dark cycle (6:00–18:00 GMT, lights on) and enough food and water was provided so that replenishment was not required for the duration of this mission.

Temperature, humidity and gas levels varied within the compartments of the Transporter and Habitat along with the ISS cabin environment due to active air exchanges between the ISS cabin and inside the Habitat (~ 0.3 m/s). In the U.S. Lab portion of the ISS where habitats were located, CO_2_ ranged from 788–4690 ppm, temperature from 21.3–24.1 °C, and humidity from 32–58% during the in-flight phase of the experiment (Sept 25 – Oct 31, 2014). Within the Habitat, sensor readings ranged from 26.3–28 C for Experimental Flight Habitats and 27.0–27.9 C for Experimental GC Habitats. The average temperatures were 26.0 °C and 26.4 °C for Experimental Flight and GC Habitats, respectively.

The Validation and Experimental Habitats on the ISS were located in different racks that housed and powered the hardware (EXpedite the PRocessing of Experiments for Space Station (EXPRESS) racks). The Validation Habitat was consistently observed to be 0.6–2.3 °C warmer than the Experimental Habitat due to differences in the local air temperature in front of the EXPRESS racks. Nonetheless, all parameters fell within the requirements for animal welfare that were set prior to the mission. The Validation and Experimental GC mice were housed in Habitats that were placed in an ISS Environmental Simulator (ISSES) chamber at Kennedy Space Center (KSC). The ISSES chambers were programmed on a 4-day delay using telemetry data from ISS to closely match the temperature, humidity, and CO_2_ partial pressure in the FLT Habitats to ensure that the GC animals on Earth were exposed to the same environmental parameters as the flight animals on-orbit, while the Validation Viv mice were housed continuously in a standard animal facility at KSC.

Transporter (Supplementary Fig. S4A): The Transporter provides for the transport of rodents on the commercial resupply service vehicle (SpaceX Dragon for this mission), both to and from the ISS. The Transporter was tested pre-flight in a series of ground-based experiments and shown to successfully house and provide life support for 20 mice (10 mice per side), which is twice the density compared to the Habitats, for up to 10 days during the ascent to the ISS or during the descent back to Earth. During transport, the Transporters were connected to the life support system provided by the Dragon vehicle. During transportation to the ISS, the animals could not be viewed or checked until they were transferred from the Transporter to the Habitats on-board the ISS by crewmembers. External sensors attached to the Transporter and Dragon recorded the environmental data during transport to and from the ISS.

Habitat: The rodent Habitat was modified from heritage flight hardware (AEM) to provide long-term housing for rodents aboard the ISS (Supplementary Fig. S4B). Differences between the AEM, developed and used for short-term Space Shuttle missions, and the Habitat include long duration and secondary exhaust filters, water refill capability, and the addition of four video cameras (Supercircuits, Austin, TX) to monitor daily activity and animals’ health and behavior. Two color cameras with wide angle lens and two black and white cameras with pinhole lens are located in the filter and lixit areas, respectively. A brief description of the different types of animal flight hardware to date is described in Table 1.

Foodbars (~850 g) developed specifically for the flight hardware^54^ were mounted onto a metal plate (two plates per Habitat, one plate per side) and approximately 2 liters of deionized autoclaved water supplemented with 4 ppm iodine (to prevent microbial growth) were installed into each Habitat. Water bags were filled and installed in the Habitats at NASA Ames Research Center (ARC) before Habitats were launched to ISS without animals. Once on-orbit, the crew members connected the Habitats to power source, installed them in EXPRESS Racks and ran a verification to ensure that telemetry (temperature, CO2, %RH), video downlink from cameras, lights, and fans are functional prior to animal transfer. Food bar plates were inserted in the Habitats prior to animal transfer on-orbit. Additionally, the Habitat allows for water refills on-orbit to support long-duration missions of more than 30 days (although water was not refilled during the mission described here).

Videos recorded from the Habitat cameras were used to assess animal health and behavior including sleeping, eating, drinking, grooming, and ambulating. The Habitat also contained sensors to detect humidity and temperature. For this mission, no additional form of enrichment was provided to the animals.

Animal Access Unit (AAU, Supplementary Fig. S4C): This unit attaches to both the Transporter and Habitat to safely access the animals during transfer between the two and for science operations, preventing escape of animal waste into the cabin environment.

Mouse Transfer Box (MTB, Supplementary Fig. S4D): A lexan box with a 2-door lid was created to hold and securely transfer up to 5 animals at a time between the Transporter and Habitat, and Habitat and Microgravity Science Glovebox, which is an experimental hood located on the ISS, that was used to euthanize the animals and dissect their tissues on-orbit.

### Pre-flight testing of rodent research hardware

Although the AEM has been successfully flown 27 times on the US Space Shuttles over the past 50 years, the Rodent Habitat has been developed by adding multiple new features as described above. Therefore, a series of ground-based tests were performed using a protocol similar to those for spaceflight (with the exception of the microgravity factor) to ensure that the Habitat could support a long-duration mission on the ISS.

#### Launch simulation test

A Launch Simulation Test (LST) was conducted to determine if the Transporter would maintain the health and well-being of mice when subjected to the SpaceX Dragon launch profile (random vibration and steady state acceleration). To do this, female C57BL/6J (14 weeks old) mice from Jackson Lab were subjected to vibration followed by centrifugation while in the Transporter to simulate the launch profile of the Dragon. For the LST the mice first were housed in the Transporter at a density of 20 mice per Transporter (10 mice per side) for 10 days to simulate the longest expected time of the transient phase between animal loading prior to launch and animal transfer on the ISS to the Habitats. At the end of the LST the mice were checked by the attending veterinarian and deemed to be healthy and suitable for experimental research. The LST was followed by a biocompatibility test in which the mice were housed in the Habitats for 32 days, similar to what was described previously for a group of young, male mice subjected to segmented bone defects^55^. For this study, a subset of mice were subject to hindlimb unloading (HU)^56^ immediately after the launch simulation test to evaluate any adverse health effects on the mice due to the sequential stressors of launch simulation and weightlessness in simulated microgravity.

#### Rodent habitat biocompatibility test

Two separate biocompatibility tests of different duration (e.g. 32 and 91 days) were performed with the Habitats to determine the length of time the Habitat can support animal health. The following was evaluated throughout the test: stability of the fans, cameras, and telemetry sensors, performance and saturation of the filters, waste buildup, and most importantly, long-term animal health and well-being. The data showed that the Habitat supported 10 female, 16 wk old C57BL/6J female mice for 32 days during the first test; there were no significant differences in body weights nor the weights of the organs that are known to respond to chronic stress (e.g. adrenal glands, thymus, and spleen) between the animals housed in standard vivarium cages and those housed in Rodent Habitats. The animals were checked by the NASA Attending Veterinarian and deemed to be healthy and suitable for science. This test was conducted using the same protocol as the RR-1 experiment described here, including the animal acclimation and daily health checks.

To assess if the Habitat can support the animals’ health and well-being for as long as 3 months, a biocompatibility test was conducted for 91 days, comparing mice housed in the Habitat continuously to mice housed in standard, shoebox cages (i.e. vivarium). The test revealed that the hardware performed as expected to support animal health (data not shown).

### Preflight preparation and transit to ISS

#### Pre-flight training of astronaut crew

Two United States Orbital Segment (USOS) crew members were trained by certified trainers in the 2-day Rodent Generic Skills class at Johnson Space Center (JSC), where they practiced and were certified for animal handling and gross dissection skills with multiple animals. The crewmembers also were trained in animal handling, euthanasia, and mission specific dissection skills using flight-like kits for tissue preservation and the Rodent Research hardware. They also received training on how to conduct a health assessment of the animals. The first priority of the training was to ensure humane animal handling and euthanasia.

#### Animals

All animal procedures performed in these experiments were approved by the Institutional Animal Care and Use Committees (IACUC) for flight at the NASA Ames Research Center (ARC) and the Kennedy Space Center (KSC) and the methods were carried out in accordance with relevant guidelines and regulations. Twelve-week old female C57BL/6J mice were purchased from the Jackson Laboratories (Bar Harbor, ME). Wild type C57BL/6NTac mice and MuRF1 (muscle RING finger protein 1) knockout (KO) mice that were generated on C57BL/6NTac background were purchased from Taconic Biosciences (Rensselaer, NY) for the Experimental cohorts, respectively. All the mice were implanted with IMI-1000 2 × 11 mm transponders (Biomedic Data Systems, Seaford, DE) prior to shipping, and were examined by the animal care facility (ACF) veterinarian staff upon arrival at KSC. Fecal samples were collected at 2 different time points: upon receipt at KSC and 11 days before turnover for launch, analyzed by Charles River Laboratories (Wilmington, MA) to verify that they are free from microorganisms before launch. The mice were housed in standard vivarium cages at a density of 5 mice per cage. The animals were provided with standard bedding, diet (LabDiet Rodent 5001), and deionized autoclaved water *ad libitum*, maintained on a 12 hr:12 hr dark/light cycle (6:00–18:00 GMT, lights on), and checked daily by the KSC and ARC staff. During the pre-flight period, body weight, food depletion, and water consumption data were collected and recorded twice a week. Animal identification and body weight measurements were collected and recorded using the Data Acquisition System (DAS-8001) and Smart Probe (SP-6004) (Bio Medic Data Systems) in conjunction with the top-loading balance (AC3102CU, OHAUS).

#### Animal pre-flight acclimation, group assignment, and flight preparation

Four days after arriving at KSC, the Validation animals were gradually acclimated to the foodbars and hardware. Twenty-two days before launch (L-22d), the mice were introduced to the NASA Type 12 Nutrient upgraded rodent foodbars (NuRFB)^54^, and at L-18d to deionized, autoclaved water via water bottles outfitted with lixits. At L-12d the mice were introduced to raised wire floors and regrouped based on their body weights, and placed at double density (10 mice per cage). Mouse Igloos (VWR, Radnor, PA) were provided to the mice for enrichment. Food and water depletion was measured, via weight changes, every 3–4 days after mice were placed at double density. Note that since foodbars crumble and fall through the wire cage grid walls during consumption, their measured weights do not accurately reflect total consumption. Similarly, Experimental mice were received at KSC at L-10 weeks (and not provided igloos as enrichment), placed on lixits, food bars and raised wire floors at L-23 days, and combined to double density at L-12 days. Loading and turnover was L-2 days.

#### Animal selection

At L-6d, three cages of the Validation mice (10 mice per cage) were selected based on similar body weights to be the Spaceflight (FLT), Ground control (GC), and Vivarium control (Viv) groups for the Validation experiment. The animals were examined by the attending veterinarian and were deemed to be healthy and suitable for the flight experiment. Experimental mice were similarly weight matched into Basal, FLT and GC groups and assessed by the AV. From these 3 groups, at L-3d, the FLT group animals were assigned based on their health and body weights. At L-2d, the FLT mice (16 weeks and 32 weeks of age for Validation and Experimental groups, respectively) were loaded into the Transporter and turned over to SpaceX for launch. The animal loading was originally scheduled to occur at L-25 hours, but due to a launch slip of ~24 hours, the mice were housed for 2 days in the Transporter prior to launch. During this time, the Transporter remained in the Dragon capsule on the launch pad.

#### Animal load and turnover for launch

The Transporters containing the FLT mice were driven to the launch pad in a temperature controlled van then turned over to SpaceX to be installed in the Dragon capsule for launch. Between turnover and loading into the SpaceX Dragon Capsule, the Transporters were connected to a power supply and kept in an air-conditioned van or room in a SpaceX facility. Data collected from sensors attached to the outside of the transporter confirmed that temperature and humidity remained within acceptable range. Once the Transporters were installed and Dragon capsule hatch closed, life support and environmental control was provided by the SpaceX Dragon. Due to a one day launch delay, the animals remained in the Transporters for two days prior to launch. To prepare for launch, the Dragon capsule atop the Falcon9 rocket transitioned from horizontal to vertical position. This rotation was replicated on the ground control Transporters. Dragon is unmanned and there is no video capability in the Transporter. As such, there were no health checks performed while animals were in the Transporter during ascent. The Dragon environmental control and life support system maintained the atmosphere within acceptable limits for pressure, temperature (65–85°F), relative humidity (25–75%), oxygen (19–23%), and carbon dioxide (≤6000 ppm). Later analysis of sensors installed in Dragon cabin confirmed that environmental conditions remained within allowable ranges.

#### On-orbit animal operations

Four days after launch (L + 4d), mice were transferred by astronauts from the Transporter to the Habitats on the ISS. As the animals were being transferred, the crew and the Attending Veterinarian (via real-time telemetry from an over-the-shoulder camera) examined the animals. All mice were deemed healthy, and they were housed at a density of 10 mice per Habitat, 5 mice per side. While on-board the ISS, animal health checks were performed daily as described below. Of the 10 Experimental mice, results from only 5 WT are reported here; muscle responses for all the Experimental mice are reported by Cadena *et al*.^14^.

Experimental mice were processed on the 17^th^ (L + 21d, 5 mice) and 18^th^ (L + 22d, 5 mice) days after animal transfer on the ISS. On these days, the mice were removed from their Habitats and brought to the MSG for handling and dissection using ventilated Mouse Transfer Boxes (MTB, Supplementary Fig. S4D). The animals were subsequently euthanized via intraperitoneal (IP) injection of Euthasol (Virbac, Fort Worth, TX) diluted with sterile saline (1:1) at 1.72 mg/kg followed by cervical dislocation then processed in the MSG on-orbit. The right hindlimb was dissected from 10 mice. The liver and spleen were collected from 5 wild-type mice and frozen at ≤−80 °C in a flight freezer (the Minus Eighty degree Laboratory Freezer for ISS, MELFI) or preserved in RNAlater (Thermo Fisher Scientific, Waltham, MA) at 4 °C for 2 days then frozen and stored at ≤−80 °C, respectively.

On the 33^rd^ day after the animals were transferred to Habitats on the ISS (37 days after launch), 10 Validation mice were euthanized via IP injection of Euthasol (1.72 mg/kg) followed by cervical dislocation then processed in the MSG. Two of the 10 mice were dissected immediately after euthanasia to recover livers and spleens on the ISS. Livers were then stored at ≤−80 °C in the (≤−80 °C) Minus Eighty-Degree Laboratory Freezer (MELFI). Spleens were dissected and preserved in RNAlater (Thermo Fisher Scientific, Waltham, MA) at 4 °C for about 2 days and then transferred to the MELFI for freezing and storage until return to Earth. The remaining 8 non-dissected Validation mice carcasses were wrapped in aluminum foil, put in Ziploc bags and stored in the MELFI (≤−80 °C). Livers and carcasses were placed in cold stowage containers that were prechilled to −130 °C to freeze the livers and carcasses in the MSG prior to transfer and storage in the MELFI.

Since only 2 of the 10 Validation mice were dissected for livers and spleens on the ISS due to crew time constraints, the liver and spleen samples dissected from 5 wild-type ISS National Lab Experimental mice were provided to NASA for post-flight analysis to increase the sample size (Fig. 2B).

The Flight Attending Veterinarian was in attendance for all on-orbit euthanasia and dissection procedures and was able to visualize and communicate with the crew via real-time video and voice loop. Each individual animal was examined and noted to be in good condition prior to euthanasia. The dissection procedures were successfully completed on all mice.

#### Baseline controls (Basal)

For both the Experimental and Validation studies, 10 baseline mice matched by pre-launch body weights to Viv, FLT and GC were housed in standard mouse cages, 5 mice per shoebox cage (11.5″ × 7.5″″ × 5″, catalog 100272 F, Lab Products, Seaford, DE) with one Igloo per cage for enrichment and maintained in an animal holding room at the KSC ACF. The mice were euthanized and processed one day after launch according to the same protocol as their respective FLT mice.

#### Ground controls (GCs)

Mice selected for GCs were housed in the Rodent hardware (Transporter and Habitats) and treated as similarly as possible to FLT animals. All procedures were off-set by a 4-day delayed schedule so that environments could be set to match telemetry data received from the ISS. Thus, GC mice were loaded into the Transporter at L + 1d. Just prior to animal loading, the mice were checked by the KSC Attending Veterinarian, and they were deemed healthy and suitable for science. The Transporter was kept in the ISSES to simulate the same environmental conditions the FLT animals were exposed to. After 4 days inside the Transporter, the Validation and Experimental GC mice were evaluated by the KSC ACF veterinarian staff and then transferred to the Habitats, where they were housed at a density of 10 mice per Habitat, 5 mice per side. The Habitats, containing the Experimental and Validation GC mice, were maintained in the ISSES for 17–18 and 33 days, respectively, under the same environmental conditions as the FLT mice. The Experimental and Validation GC mice were dissected and processed using the same timelines (off-set by 4 days) and protocols as their respective FLT mice.

#### Vivarium controls (Viv)

The Validation mice selected for the Viv group were housed in standard mouse cages, 5 mice per shoebox cage with one Igloo per cage for enrichment and maintained in an animal holding room at the KSC ACF. The KSC ACF veterinary staff performed health checks daily, and weighed the mice, food, and water twice weekly and their food and water were replaced once weekly. The Validation Viv mice were dissected and processed using the same timelines and protocols as their respective FLT and GC mice.

#### Video health checks

Daily health checks were performed for Experimental and Validation FLT and GC animals (health assessments were performed on Viv animals as described in the Vivarium control section above) by trained ground personnel beginning one day after the animals were transferred from the Transporter to the Habitats (GMT 269, September 26, 2014). Initially, 2 hours of video (one hour each during the light and dark cycles) were recorded from each Habitat. Two cameras were located in each side of the Habitat - one near the lixits and the other near the filter. These videos were downlinked to Ames Research Center (ARC, Moffett Field, CA) via Marshal Flight Space Center (MFSC, Huntsville, AL), and animal health checks were performed by observing and documenting the health and behaviors of the animals for 1 hour during the light cycle and 1 hour during the dark cycle, daily from each Habitat. However, due to thermal issues experienced with this recording schedule, the video health checks for the Validation mice were shortened to approximately 15 minutes. Starting on Oct 10, 2014 (GMT 283), the health checks during the light cycle were discontinued with concurrence from the Institutional Animal Care And Use Committee (IACUC) and for the remainder of the mission, real-time health checks were performed during the dark cycle only.

Animal health check forms were used to record daily observations of the following: Habitat ID, Habitat Side, Camera position, Health Status, GMT time/date, number of animals observed, animal cycle, and animal behaviors (drinking, eating, grooming, ambulation, etc.). Animal activity was recorded during the health check on a scale of 1–3 based on different behaviors as described in Fig. 3A. To further evaluate animal behavior on-orbit, one 24-hour video monitoring session was performed in which the animals were observed for 10 minutes every hour.

#### Radiation exposure

The RR-1 Validation mice were exposed to radiation for 37 days (4 days in transit and 33 days on the ISS). The dosimetry mapping of radiation levels across the U.S. Lab indicated that during the 37 day period the daily dose was approximately 207 μGy/day and the total dose recorded by an instrument in the U.S. lab was 7.377 mGy. This range falls within expected and allowable radation doses. In addition, the Rodent Habitats were inside a locker and inserted into the EXPRESS racks, which provided additional shielding to the mice. Radiation doses during transit to the ISS were not determined; however, since the mice remained inside the radiation belts during transit, we estimated that the dose rate during transit was approximately the same as while on board the ISS, which would be 0.6 to 0.9 mGy during transit.

#### Food and water depletion

To provide an estimate of water and food consumption for comparison between FLT and control groups, foodbars and water boxes from their respective cages were weighed before launch and after return to Earth. Since the mice may drain some water from the lixits and cause foodbars to crumble without being consumed, results are reported as food and water depletion (rather than consumption) and normalized to the number of mice in each compartment per day (n = 2 measurements/group).

#### Post-flight sample return and processing

Experimental and Validation FLT frozen tissues, carcasses, and remaining food and water were returned to Earth 12–13 and 105 days after dissection, respectively, and all frozen samples were kept at ≤−70 °C during transport. All Experimental and Validation Basal, GC, and Viv (Validation only) frozen tissue samples and carcasses were shipped from KSC to ARC on dry ice then stored at −80 °C until they were processed about 4 months later.

Frozen carcasses of the Validation mice were weighed upon receipt at ARC, and the body weights of the 8 non-dissected mice from each group were compared to their respective weights at L-3 days. Tests performed prior to launch had confirmed that the long term freezing and partial thawing procedure did not affect measured body weights (data not shown).

After brief (~5 min), partial thawing at room temperature, disection was initiated for the following organs until all tissues were complete about ~35–45 min after removal from the freezer. Immeidately after dissections, tissues and were weighed, and then preserved by storing in RNAlater: gastrocnemius, soleus, tibialis anterior (TA), extensor digitorum longus(EDL), quadriceps, liver (part of the liver was also frozen in liquid nitrogen then stored at −80 °C to be used in enzyme activity assays described below), spleen, thymus, and adrenal glands. Additionally, 3 mice that were the same sex, strain, and age as the Validation mice (female C57BL/6J, ~21 weeks at time of euthanasia) were euthanized by IP injection with Euthasol followed by cervical dislocation, then select tissues were dissected and prepared in the same manner as the Validation mice except under optimal laboratory conditions, i.e. aliquots of livers were immediately frozen post-dissection in liquid nitrogen for the enzyme assays, and spleens and aliquots of livers were immediately preserved in RNAlater, to serve as a positive control for tissue quality. Tissues were stored at −80 °C immediately after freezing in liquid nitrogen, and tissues preserved in RNAlater were kept at 4 °C for 2 days then frozen and stored at −80 °C.

#### RNA isolation and evaluation

RNA was extracted from the spleen and liver tissue samples from Validation Basal, Viv, GC, FLT, and positive control (PC) mice, and from the ISS National Lab 5 wildtype mice using TRIzol reagents (Thermo Fisher Scientific, Waltham, MA). The RNA samples were then treated with DNase I incubation mix (2.727 Kunitz units/uL; RNase-free DNase kit, Qiagen Catalogue #79254), and RNA concentration and purity were assessed using a NanoDrop 2000 UV-Vis Spectrophotometer (Thermo Fisher Scientific, Waltham, MA). RNA quality was determined by measuring the RNA integrity number (RIN) using the Bioanalyzer 2100 (Agilent Technologies, Santa Clara, CA), where a RIN of 10 is considered intact and 0 totally degraded RNA. The analysis was performed according to the manufacturer’s instructions.

The total RNA extracted for each tissue sample was converted to cDNA using the High-Capacity cDNA Reverse Transcription Kit (Thermo Fisher Scientific Catalog # 4368814) according to the manufacturer’s protocol. TaqMan Gene Expression Assay primer and probe sets (Thermo Fisher Scientific) were used for real-time, quantitative PCR (RTqPCR) analysis of *Hmgcr* (Mm01282491_g1), *Rpl19* (Mm02601633_g1), *Gapdh* (Mm99999915_g1), *Cat* (Mm00437992_m1), *Gsr* (Mm00439154_m1), *Serpina1e* (Mm00833655_m1), *Cdkn1a* (Mm00432448_m1), *Chka* (Mm00442759_m1), *Pnpla3* (Mm00504420_m1), *Tef* (Mm00457513_m1), *Snd1* (Mm00490722_m1), *Nr4a1* (Mm01300401_m1), *Nox4* (Mm00479246_m1), and *Taf3* (Mm01337577_m1). RTqPCR analyses were carried out using 20 ng of cDNA on a QuantStudio 6 Flex Real-Time PCR System (Thermo Fisher Scientific Catalog #4485694). Gene expression in liver samples was normalized to the *Rpl19* (all but *Cat*) or *Gapdh* (*Cat*) endogenous reference genes. All data are expressed as 2^−(Ct(target)-Ct(endogenous reference))^.

### Liver enzyme activity assays

Protein was extracted from an aliquot of each liver sample from Basal, GC, and FLT Experimental mice (n = 5 from each group), and from Basal, Viv, GC, and FLT Validation mice (n = 2 for the tissues that were dissected either on the ground or on-orbit then frozen; and n = 5 (out of 8) for the samples that were dissected from frozen carcasses from each group), as well as their respective positive controls (n = 5 for Experimental control and n = 3 for Validation control). Activities of three enzymes were determined using the method previously described^57^. Briefly, catalase activity was measured using the colorimetric assay according to the manufacturer’s instructions (Oxford Biomedical Research, Oxford, MI), and normalized to total protein levels (Thermo Scientific™ Pierce™ Micro BCA™ Protein Assay Kit, Pierce Biotechnology, Rockford, IL). Catalase protein levels were measured using the Catalase Human ELISA Kit (Abcam, Cambridge, MA), and specific activity of the enzyme was calculated (U/μg catalase protein). GSR activity levels were determined using the GSR Assay Kit (Abcam, Cambridge, MA) and GAPDH activity levels were determined using the colorimetric GAPDH assay kit (ScienCell Research Laboratories, Carlsbad, CA).

### Statistical analysis

Statistical significance was determined by comparing 4 and 3 groups from the same cohorts for the Validation and Experimental study, respectively; for the Validation mice, these included the FLT, GC, VIV and Basal groups and for Experimental mice these included FLT, GC and Basal groups. For Validation mice, uniformity of variance was tested by Levene’s test and normality goodness of fit by Shapiro-Wilk. One-factor ANOVA was then applied to the data, and if the main effect showed P < 0.05, was followed by a Tukey-Kramer HSD post hoc analysis. The sole exception to application of ANOVA was Validation liver catalase activity, which did not display normal distribution. The non-parametric Kruskal-Wallis test was applied to Validation catalase results. P < 0.05 was accepted as significant. All data shown are mean +/− S.D. Data analyses were performed using JMP13.1 (SAS).

## Supplementary information

Supplementary information.
