# Supplementary material for: Validation of a New Rodent Experimental System to Investigate Consequences of Long Duration Space Habitation
Source: Sci Rep. 2020 Feb 11;10:2336. doi: 10.1038/s41598-020-58898-4 (PMC7012842; doi:10.1038/s41598-020-58898-4)
Supplement: Supplementary file 1 — Supplementary information. [file 41598_2020_58898_MOESM1_ESM.pdf]

VALIDATION OF A NEW RODENT EXPERIMENTAL SYSTEM TO INVESTIGATE CONSEQUENCES OF LONG DURATION SPACE HABITATION

Sungshin Choi[1], Amanda Saravia-Butler[2], Yasaman Shirazi-Fard[3], Dennis Leveson-Gower[1], Louis S. Stodieck[4], Samuel M. Cadena[5], Janet Beegle[3], Stephanie Solis[6], April Ronca[3,7], Ruth K. Globus[3]*

[1]KBRwyle, NASA Ames Research Center, Moffett Field, CA
[2]Logyx, LLC, Mountain View, CA
[3]Space Biosciences Division, NASA Ames Research Center, Moffett Field, CA
[4]BioServe Space Technologies, Department of Aerospace Engineering Sciences, University of Colorado, Boulder, CO.
[5] Novartis Institutes for Biomedical Research, Cambridge, MA
[6] LifeSource Biomedical Services LLC
[7] Department of Obstetrics & Gynecology, Wake Forest Medical School, Winston-Salem, NC

*corresponding author

Supplementary Figure S1

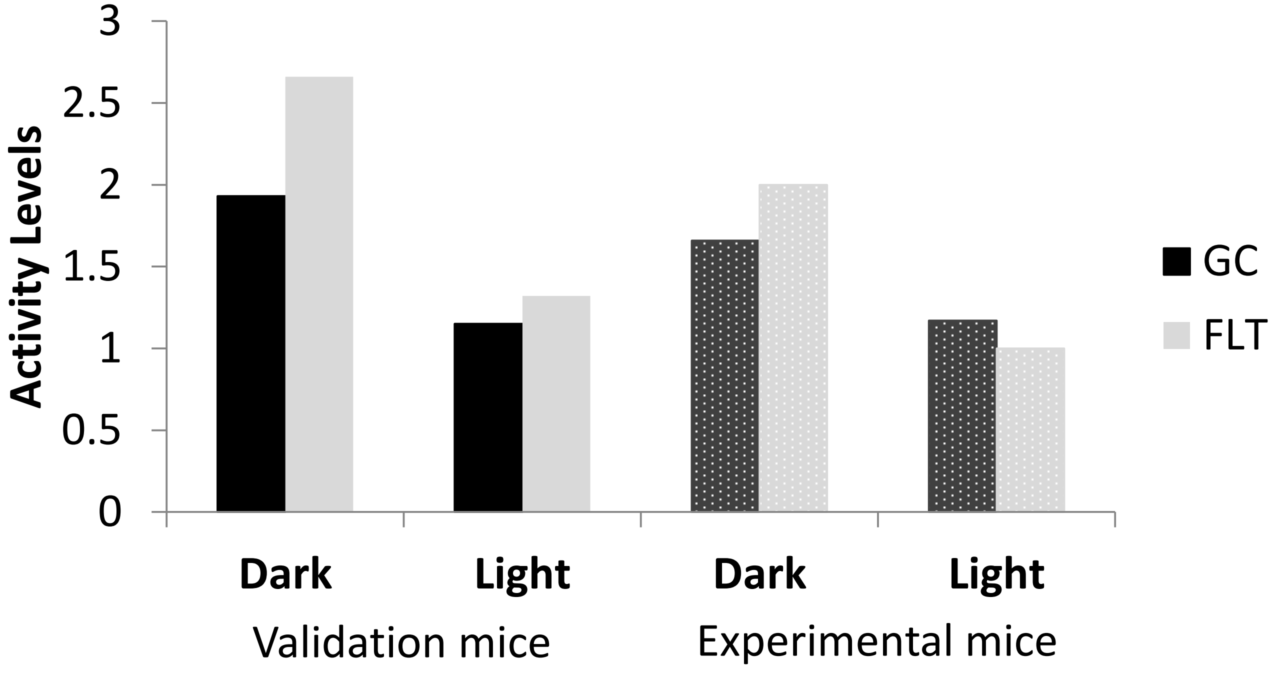

Supplementary Figure S1. Spaceflight increased animal activity. Average activity scores for mice in each side of the Habitat for Flight and Ground Control groups in the Validation and Experimental studies. . N=2 Habitat sides (containing 5 mice each) for each group.

Supplementary Figure S2

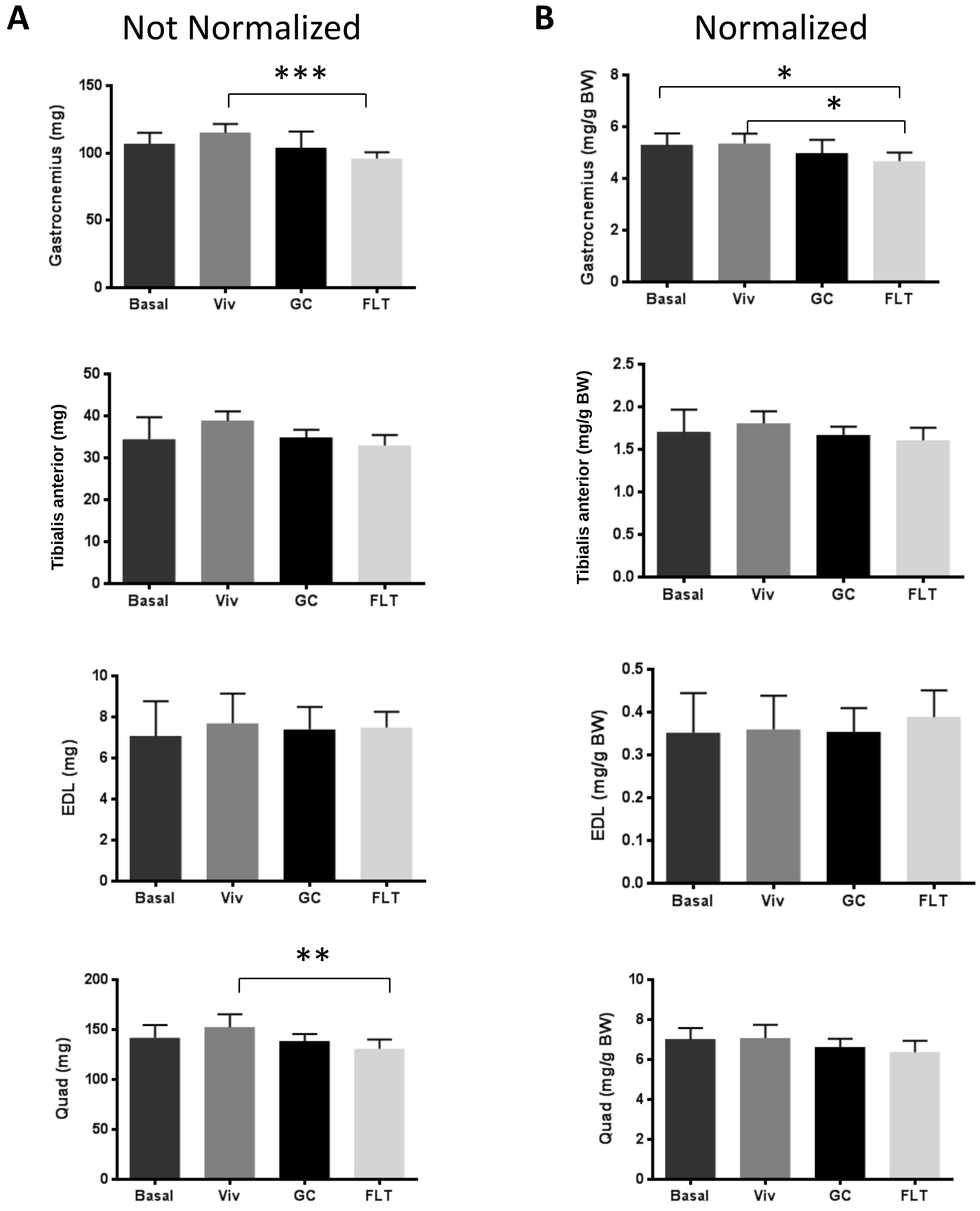

Supplementary Figure S2

Supplementary Figure S2. Spaceflight did not cause decrements in muscle mass (other than the soleus). The indicated muscles were dissected from the frozen carcasses of all Validation groups, thawed and weighed according to methods. Raw weights of each muscle (A), and muscle weights after normalization to body weight (B) were quantified. N=8 for each group. *$p<0.05$; **$p<0.01$; ***$p<0.001$.

Supplementary Figure S3

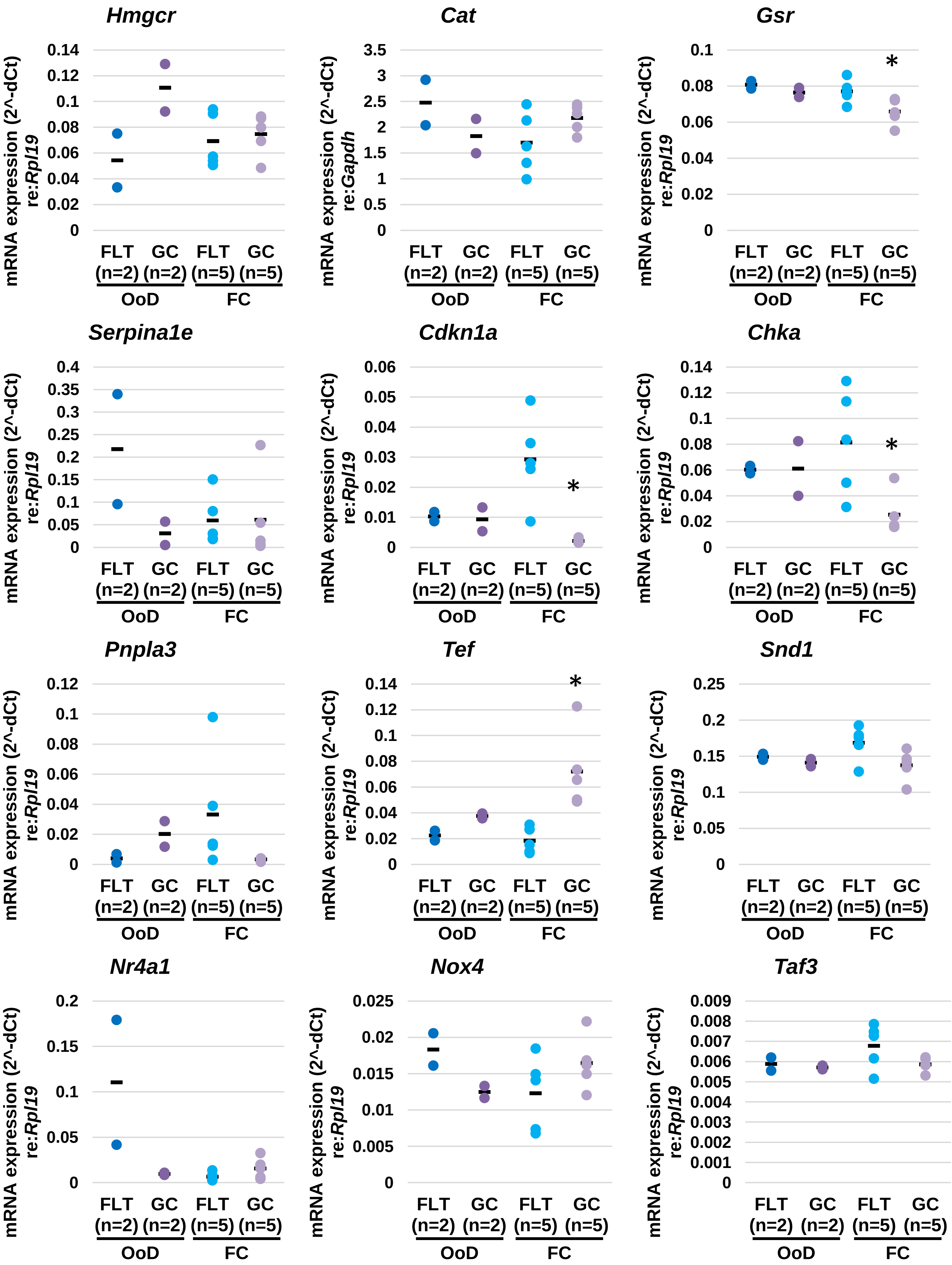

Supplementary Figure S3

Supplementary Figure S3. Expression of select liver-associated genes. The expression of select genes was determined using RNA extracted from Validation mouse livers dissected on-orbit (or on the ground) and frozen at -80°C until recovery (On-orbit Dissected, OoD) and from frozen carcasses that were returned to Earth (Frozen Carcasses, FC) using RTqPCR. Values were normalized to *Rpl19* or *Gapdh* expression and are expressed as 2^-ΔCt. N numbers are indicated. * indicates $P<0.05$ by ttest comparing FLT to GC in FC tissues.

Supplementary Figure S4

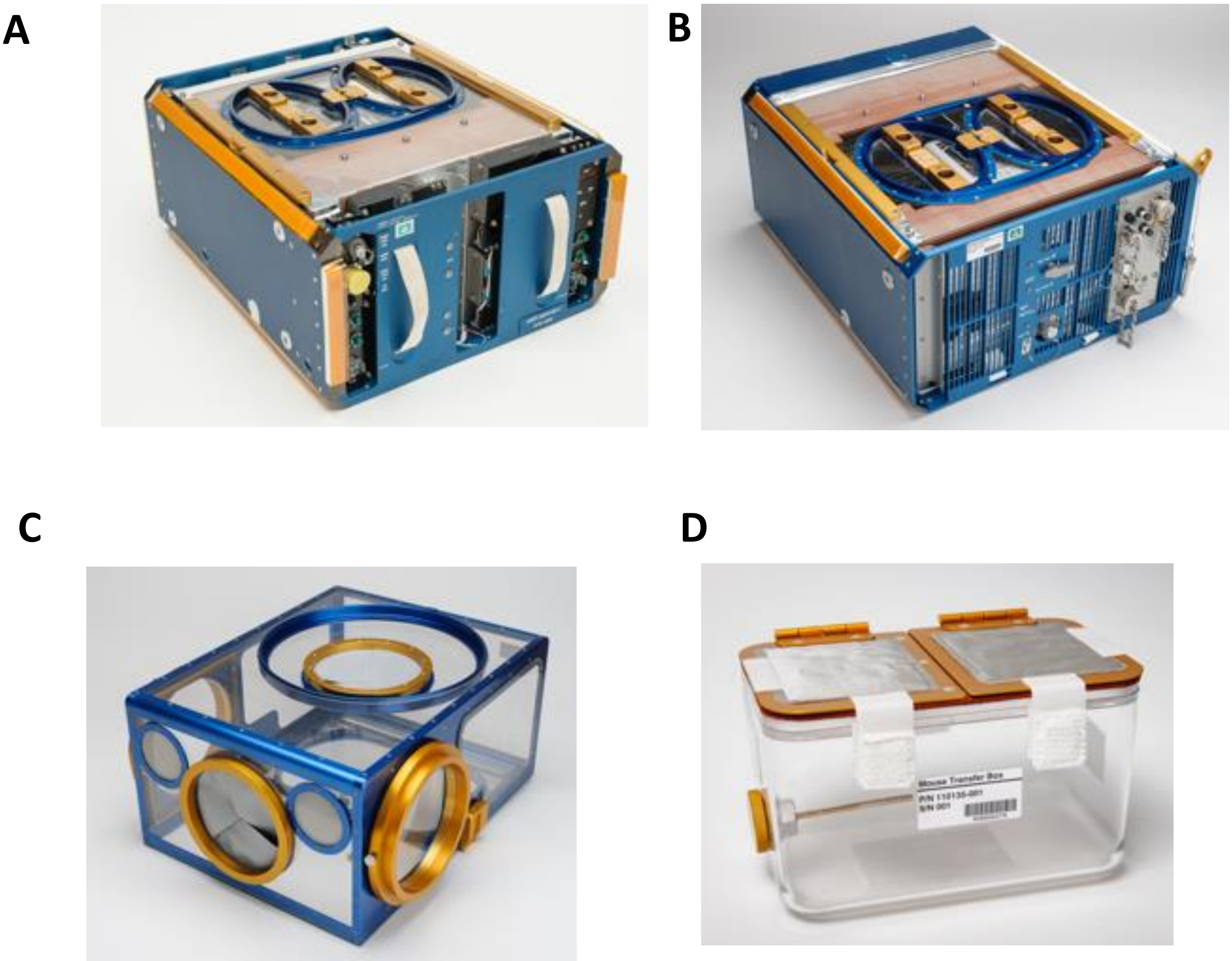

Supplementary Figure S4. Rodent Research 1 Hardware. A) Transporter, B) Habitat, C) Animal Access Unit. D) Mouse Transfer Box. Images are not to scale.

Supplementary Table S1

Activity	Experiment Timeline Relevant to Launch
Arrival and acclimation of the mice	L-27 days (arrival); L-25 hours (loaded into the Hardware)
Load mice into Transporter	L-2 days
Load into Dragon	L-2 days
Launch	L
Dragon dock with ISS	L+2 days
Mice transferred to Habitats on ISS	L+4 days
Experimental mice:<br>on-orbit euthanasia and dissection<br>Return of samples to Earth	 L+21 and L+22 days<br>L+35 days
Validation mice:<br>on-orbit euthanasia and dissection<br>Return of samples to Earth	 L+37 days<br>L+140 days
Tissue Analysis of Validation mice	L+8 to 14 months

Supplementary Table S1. Timeline of activities for the RR-1 mission.

Supplementary Table S2

		Validation Mice	Experimental Mice
Foodbar (g/mouse/day)	VIV	3.90	N/A
	GC	3.06	4.40
	FLT	3.53	5.70
Water (g/mouse/day)	VIV	2.17	N/A
	GC	2.94	2.90
	FLT	5.81	4.90

Supplementary Table S2. Spaceflight caused increased water and food depletion. Water and foodbars in FLT and GC cages were weighed before and after completion of the Experimental and Validation studies and the depletion per mouse per day was calculated. An average of n=2 per group is shown.
